# Validation of sampling methods in bulk feed ingredients for detection of swine viruses

**DOI:** 10.1111/tbed.13326

**Published:** 2019-10-21

**Authors:** Cassandra Jones, Savannah Stewart, Jason Woodworth, Steve Dritz, Chad Paulk

**Affiliations:** ^1^ Department of Animal Sciences and Industry Kansas State University Manhattan Kansas; ^2^ Department of Diagnostic Medicine/Pathobiology Kansas State University Manhattan Kansas; ^3^ Department of Grain Science and Industry Kansas State University Manhattan Kansas

**Keywords:** bulk, diarrhoea, epidemic, feed, ingredient, porcine, sampling, sensitivity, virus

## Abstract

Animal feed can be contaminated with fomites carrying swine viruses and subsequently be a vehicle for viral transmission. This contamination may not be evenly distributed, and there is no validated sampling method for detection of viruses in animal feed or ingredients. The purpose of this experiment was to evaluate the sensitivity of ingredient sampling methods for detection of porcine epidemic diarrhoea virus (PEDV). No animals were used in this experiment, so approval from an animal ethics committee was not necessary. Thirteen kg soybean meal was used in a 2 × 2 factorial plus a control, with 2 doses of PEDV (Low: 10^3^ TCID_50_/g versus High: 10^5^ TCID^50^/g) and two sample types (individual probes versus composite sample). Soybean meal was confirmed PEDV negative, then loaded into individual, 1‐kg polyethylene tote bags with PEDV introduced after loading the first 100 g. There were six replicates per PEDV dose plus a control. Ten individual probes or one composite sample per bag were created and analysed for PEDV via qRT‐PCR. The interaction, dose and sample type were significant for both PEDV presence and quantity. No control samples had detectable PEDV. At the low dose, no PEDV RNA was detected in individual probes or composite samples, but was confirmed in 100% (32.4 C*_t_*) of the inoculant samples. This is likely due to loss of sensitivity during the analysis process, which has been previously reported to cause a loss up to 10 C*_t_* when detecting PEDV in feed or ingredients. At the high dose, only 37% (37.7 C*_t_*) of the probes had detectable PEDV RNA. Composite samples were more sensitive (*p* < .05), with PEDV RNA detected in 100% of samples (35.7 C*_t_*). In summary, sampling bulk ingredients for PEDV should include compositing at least 10 individual samples. Future research is needed to identify alternative methods that have a similar sensitivity, but require less time and effort to collect such a sample.

## 1 INTRODUCTION

Controlled research has demonstrated the ability for many domestic and emerging transboundary swine viruses to survive in ingredients when exposed to conditions mimicking those of transboundary shipment (Dee et al., 2018, 2016). Furthermore, both porcine epidemic diarrhoea virus (PEDV; Schumacher et al., 2016) and African swine fever virus (ASFV; Niederwerder et al., 2019) contamination in feed has been demonstrated to cause animal illness in research settings. Epidemiological evidence also points to the animal feed supply chain being the most likely source of PEDV entry into the United States (USDA‐APHIS, 2015) and a source of subsequent transmission throughout North America (Aubry, Thompson, Pasma, Furness, & Tataryn, 2017; Dee et al., 2014; Pasick et al., 2014). Sampling feed or ingredients for viral contamination would be a valuable screening tool to either limit domestic virus spread or prevent entry of a foreign animal disease. However, there is no validated method for sampling viral contamination in bulk animal feed or ingredients. This limits the confidence pork producers and feed manufacturers have in ingredients, testing PCR‐negative for domestic viruses, and is a key justification for the inability to conduct foreign animal disease surveillance in imported ingredients (USDA‐APHIS, 2019).

**Figure 1:**
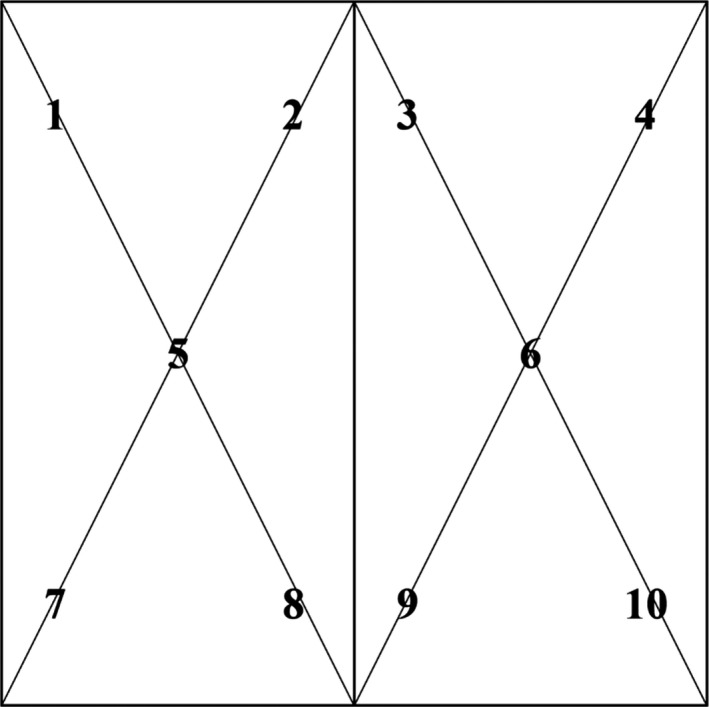
Bulk ingredient sampling locations. Example, bulk ingredient sampling locations for detection of contaminants that may not be evenly distributed. Each number represents a location of a probe at varying depths, with samples combined to create a single composite sample

The appropriate method for sample collection varies with the type and distribution of a substance. For example, most United States feed mills collect a single grab sample from a 23,500‐kg semitrailer or 1‐metric ton polyethylene tote bag. This is typically sufficient, because the purpose of the sample generally to analyse nutrient characteristics, which vary little throughout a single lot of ingredient. However, some contaminants are known to occur in ‘hot spots’ instead of being evenly distributed. This is the case with aflatoxin, where conditions in a particular portion of a field may contribute to high concentrations of the contaminant in some grain kernels, with no contamination in others. When the FDA action limit for aflatoxin in some species, such as lactating dairy cows, is as small as 20 ppb (FDA‐CVM, 2019), more sensitive sampling is necessary to detect the miniscule quantity in a large volume of bulk material. The Association of American Feed Control Officials (AAFCO) Feed Inspector's Manual, which is used by federal and state FDA investigators when collecting samples, suggests collecting ten probes of the bulk ingredient through two ‘x’ patterns (AAFCO, 2014), as shown in the example in Figure 1. The ten probe samples are then mixed together to create a single composite sample, which has a greater probability of detecting contamination that may not be evenly distributed. It is more time consuming and extensive than a single grab sample, but this method is hypothesized to be appropriately sensitive for detecting swine viruses, which may also be located in ‘hot spots’ where faeces or other fomites are comingled with the feed or ingredient. However, it has never been validated for detection of viruses. Therefore, the objective of this experiment was to evaluate the sensitivity of ingredient sampling methods for detection of porcine epidemic diarrhoea virus (PEDV).

## 2 MATERIALS AND METHODS

Neither humans nor animals were used as research subjects in this experiment, so relevant approvals were not applicable.

### 2.1 General

This experiment was conducted in a biosafety level‐2 hood at the Kansas State University Veterinary Diagnostic Laboratory as a 2 × 2 factorial plus a control, with 2 doses of PEDV (Low: 10^3^ TCID_50_/g vs. High: 10^5^ TCID^50^/g) and two sample types (individual probes vs. composite sample). Thirteen kg of soybean meal (48% crude protein) was secured from a United States source, confirmed to not contain detectable PEDV RNA and allocated into 1‐kg lots. A new polyethylene tote bag capable of holding 1 metric ton (Uline, Pleasant Prairie, WI) was used to construct 13 individual tote bags capable of holding 1 kg. One bag was filled with 1 kg soybean meal as the control.

### 2.2 Inoculation and Sampling

Three g of soybean meal was removed from each of the remaining 12 lots of soybean meal and inoculated with either a low (10^3^ TCID_50_/g) or high (10^5^ TCID_50_/g) level of PEDV. The viral inoculum was derived in cell culture USA/IN/2013/19338, passage 8. From this, a 0.5 g subsample was reserved for subsequent analysis, while the remaining 2.5 g served as the contaminant. The remaining 997 g of soybean meal in each lot was added to the individual, 1‐kg polyethylene tote bags, with approximately 100 g added to each bag, followed by the 2.5 g of inoculant, followed by the remaining 887 g of soybean meal. There were six replicates per PEDV dose plus a control. Product within bags was then probed in ten different locations of varying depth as depicted in Figure 1, with individual sampling probes that collected approximately 1 g of sample per probe. Each probe sample was divided, with approximately 0.5 g being reserved for subsequent analysis and the remaining 0.5 g reserved for the composite sample. Composite samples were created by combining the ten 0.5‐g reserved samples from each lot and handshaking in a sealed container for thorough mixing. Loading and sample collection was initially completed for the control sample, followed by the replicates with a low dose of PEDV, and finally replicates with a high dose of PEDV. The contaminant, individual probe and composite samples were then submitted to the Kansas State University Veterinary Diagnostic Laboratory for PEDV analysis as described by Gebhardt et al. (2016) via qRT‐PCR, with a level of detection at 40 cycle threshold (C*_t_*).

### 2.3 Statistical analysis

A level of 40 was used when there was no detectable PEDV RNA. Data were analysed using the GLIMMIX procedure of SAS Institute, Inc, with the main effects of PEDV dose and sample type, as well as their interaction. Data were considered significant if *p* < .05. Degrees of freedom were approximated using Kenward–Roger, and the LSMEANS procedures and LINES option were used to separate means that differed at *p* < .05.

## 3 RESULTS AND DISCUSSION

Feed, ingredients, and their delivery were partially responsible for the rapid and widespread of PEDV throughout North America in 2013–2014 [6, 7 8]. As African swine fever virus, classical swine fever virus, and foot and mouth disease virus continue to spread among our global trade partners, it is vital their entry into the United States is prevented. Feed and ingredients are considered risks for foreign animal disease entry into non‐endemic regions because their contamination would likely lead to multiple exposures to domestic animals. As described recently in Niederwerder et al. (2019), the ASFV infection risk with contaminated feed is relatively low for a single exposure (10^4^ TCID_50_/g for one meal of 100 g of feed). However, it is likely that contaminated feed would instead be fed to multiple pigs housed in the same environment and consuming multiple contaminated meals, which substantially lowers the dose of ASFV needed to result in a similar probability of infection (10^0^ TCID_50_/g for 30 meals of 100 g of feed). Routine surveillance of imported ingredients is necessary to better understand risk of various ingredient types from other countries. However, this type of surveillance is currently neither performed nor allowed by our regulatory agencies because there is no validated sampling, extraction and detection method available at this time. This study used PEDV, a biosafety level‐2 pathogen as a model for viral contamination within an ingredient as PEDV, and the aforementioned foreign animal diseases are most likely to contaminate ingredients through faecal contamination. A ‘hot spot’ contaminant model was selected within the bulk ingredient because faecal contamination is most likely to occur within a single location prior to processing, as opposed to being evenly distributed throughout a batch or lot of product. Soybean meal was selected as the represented ingredient due to its likelihood of import from countries with circulating foreign animal disease, as well as the demonstrated ability for the same viruses to have a relatively high stability in soybean meal compared to other ingredients (Dee et al., 2018, 2016).

Individual probes and the composite sample from the control were confirmed to not contain detectable PEDV RNA. Sample type, PEDV dose and their interaction all significantly impacted (*p* < .05) the prevalence and mean quantity of detected PEDV. The contaminant used to introduce a low or high dose of PEDV into soybean meal was confirmed to contain PEDV in all 6 replicates within each dose, with an average qRT‐PCR cycle threshold (C*_t_*) of 32.4 for the low dose, which was greater (*p* < .05) than the 22.3 for the high dose (Table 1). These C*_t_* are similar to those reported by Schumacher et al. (2016) for a low PEDV contamination in feed (33.2 C*_t_* for 10^3^ TCID_50_/g) and by Cochrane, Dritz, Woodworth, and Jones (2015) for a high level of PEDV contamination in feed (22.9 for 10^3^ TCID_50_/g).

**Table 1:** Impact of sample type on sensitivity of detecting porcine epidemic diarrhoea virus (PEDV) prevalence and mean quantity in soybean meal as determined by qRT‐PCR.

PEDV Dose	Contaminant	Individual probes	Composite sample	*SEM*
Prevalence of samples containing PEDV, %				
Control	–	0 (0/10)	0 (0/1)	–
Low (10^3^ TCID_50_/g)	100^a^ (6/6)	0^c^ (0/60)	0^c^ (0/6)	12.7
High (10^5^ TCID_50_/g)	100^a^ (6/6)	37^b^ (22/60)	100^a^ (6/6)	12.7
Mean quantity of PEDV, C*_t_*				
Control	>40	>40	>40	–
Low (10^3^ TCID_50_/g)	32.4^d^	>40^a^	>40^a^	0.85
High (10^5^ TCID_50_/g)	22.3^e^	37.7^b^	35.7^c^	0.85

Superscript letters means within response criteria in the table that do not share a common superscript differ *p* < .05.

At the low dose, neither the individual probes nor the composite samples had detectable PEDV RNA. This is potentially due to the challenges of recovery and stability of viral nucleic acids in ingredients. It has been well established that 10 to 13 C*_t_* in sensitivity are lost when moving from an inoculant into a dry feed matrix (Schumacher et al., 2016, Gebhardt et al., 2016, Cochrane et al., 2015, and Cochrane et al., 2017). Of particular concern is that the 32.4 C*_t_* in the contaminant is below the minimum infectious dose for PEDV (Schumacher et al., 2016). This suggests that a poor viral nucleic acid extraction recovery may lead to a false negative in a qRT‐PCR analysis of an ingredient, but that the consumption of the ingredient may actually lead to animal illness. Schumacher et al. (2019) reported this occurrence, where a PCR‐negative sample of feed led to PEDV infectivity in a swine bioassay (Schumacher et al., 2019). As described by the United States Department of Agriculture, the extraction process of viral nucleic acids from feed and ingredients is urgently needed to improve the ability to detect low levels of virus (USDA‐APHIS, 2019) and thereby prevent false negative results that may lead to inadvertent animal disease entry.

When a high dose of PEDV was used to contaminate soybean meal, only 37% of the individual probes contained PEDV (22 of 60 total samples), with an average C*_t_* of 37.7. Comparatively, the composite sample had greater (*p* < .05) sensitivity as 100% (6 of 6 total samples) were positive for PEDV RNA, with an average C*_t_* of 35.7. These results are supported by previous research, which report sampling methods that are capable of detecting unevenly distributed contaminants in bulk ingredients. In particular, both *Salmonella* spp. (Jones & Richardson, 2004) and aflatoxin (Johansson, Whitaker, Giesbrecht, Hagler, & Young, 2000) contamination in bulk ingredients are reported with high prevalence when using a sampling method similar to that used herein. While the creation of a composite sample from 10 sub‐samples is clearly a more sensitive method for viral detection in a bulk ingredient, it also takes more than 10 times longer and more effort to generate than a single sample.

A limitation of this experiment is that the research was conducted at laboratory scale in 1‐kg totes instead of 1‐metric ton totes, bulk trucks, rail cars or vessels. However, scale was reduced to be consistent in all manners. For example, the size of each individual probe and resultant composite sample was based on the collection of 0.05% of the total sample for analysis. This is the same percentage generated when FDA investigators sample large scale bulk feeds and ingredients (AAFCO, 2014).

In conclusion, analysing a single sample of a bulk ingredient is not a reliable or sensitive method for detecting swine viruses. However, swine viruses can be accurately detected in bulk ingredients by collecting at least 10 evenly distributed samples representing 0.05% of the volume of the bulk ingredient and subsequently creating a single composite sample for analysis. Unfortunately, this requires substantial time and effort. Additional research is needed to identify alternative sampling methods that have a similar sensitivity, but with greater efficiency. A key component to this is to validate the sample preparation, extraction and detection of nucleic acids in feeds and ingredients. In the interim, it is crucial to expend the necessary effort to collect a representative product sample so accurate decisions can be determined about an ingredient's potential safety or risk.
